# Case Report: A rare coexistence with severe aortic root dilatation and nutcracker phenomenon in pediatric Marfan syndrome

**DOI:** 10.3389/fped.2026.1790656

**Published:** 2026-04-14

**Authors:** Xiaoyu Qiao, Yanyu Chen, Danyan Su, Lifeng Shang, Yusheng Pang

**Affiliations:** 1 Department of Pediatrics, The First Affiliated Hospital of Guangxi Medical University, Nanning, Guangxi, China; 2 Difficult and Critical Illness Center, Pediatric Clinical Medical Research Center of Guangxi, Nanning, Guangxi, China; 3 Guizhou Branch of Shanghai Children’s Medical Center, Guizhou, China; 4 Guizhou Provincial People’s Hospital, Guizhou, China

**Keywords:** aortic root dilatation, case report, Marfan syndrome, nutcracker phenomenon, pediatrics

## Abstract

Marfan syndrome (MFS) is a multisystem connective tissue disorder affecting the cardiovascular, ocular, and skeletal systems. We report a case of a 13.5-year-old boy who presented with excessive linear growth. Diagnostic evaluations revealed severe aortic root dilatation, repeatedly positive occult blood in urine, and ultrasonographic findings suggestive of left renal vein entrapment. Genetic testing identified a pathogenic variant in the FBN1 gene. The patient was ultimately diagnosed with Marfan syndrome complicated by left renal vein entrapment syndrome (nutcracker phenomenon). The co-occurrence of severe aortic root dilatation and left renal vein entrapment syndrome in childhood Marfan syndrome is relatively uncommon. This case may provide valuable insights for clinical diagnosis and management.

## Introduction

Marfan syndrome (MFS) is a rare autosomal dominant connective tissue disorder caused by mutations in the FBN1 gene, which encodes fibrillin-1, a key structural component of the extracellular matrix that provides support to connective tissues, especially in the arterial wall, ocular structures, and other supportive tissues. The global prevalence is estimated to be between 1/5,000 and 1/10,000 individuals (1). The clinical presentation of MFS is highly heterogeneous, spanning a broad phenotypic spectrum from mild forms with only one or a few systems involved to severe, rapidly progressive multisystem disease (2). Cardiovascular involvement represents the most serious manifestation of MFS, with aortic aneurysm and dissection being the leading causes of death, particularly among older children and adults (3). Although no large-scale cohort studies have yet explored the relationship between Marfan syndrome and nutcracker syndrome (NCS), cases of concurrent nutcracker phenomenon and severe aortic root dilation have been rarely reported in pediatric patients.

## Case description

### Clinical data

This case report was followed the CARE guidelines. The patient is a 13-year and 6-month-old boy who was admitted to the First Affiliated Hospital of Guangxi Medical University on June 19, 2020, due to “excessive height growth for over two years”. The excessive growth began more than two years prior to admission. His height was 165 cm in March 2018 and increased to 183 cm by April 2020, representing an 18 cm growth over two years. This was accompanied by flexion contractures of the toes, preventing full extension, and decreased visual acuity. Past medical history includes an inguinal hernia repair performed at a local hospital at approximately 2 years of age. Other aspects of his past history, personal history, and family history were unremarkable.

**Figure 1:**
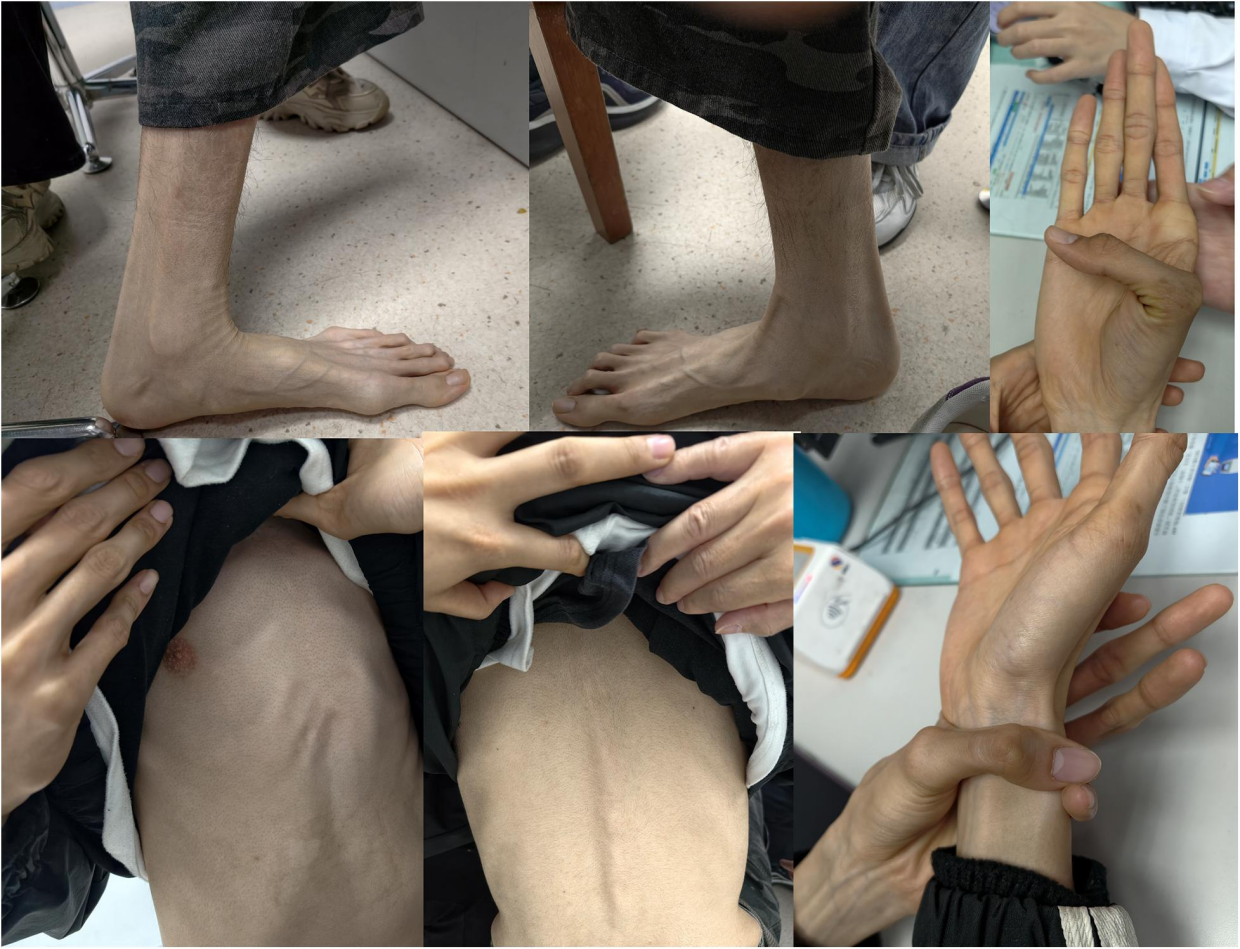
Abnormal physical examination on the chest, back, and limbs.

Physical examination on admission: Temperature 36.5 °C, pulse 92 beats/min, respiratory rate 20 breaths/min, blood pressure 108/65 mmHg, height 188 cm, weight 53.3 kg (Table 1). He presented with a tall, slender build and distinctive facial features including macrocephaly and micrognathia. Striae atrophicae were observed over the body. The chest showed pectus carinatum (Figure 1). Lung auscultation revealed clear breath sounds without rhonchi or rales. The cardiac apex was displaced downward and to the left. Heart rhythm was regular with strong heart sounds, and no murmurs were audible over any valve area. Scoliosis, arachnodactyly (long, slender fingers and toes), and flexion contractures of the toe joints were noted. Muscle tone in all four limbs was normal. Abdominal and neurological examinations were unremarkable.

**Table 1:** Relevant clinical and imaging data.

Parameter	Value
Demographics and physical exam	
Age at admission	13 years 6 months
Height	188 cm
Weight	53.3 kg
Blood pressure	108/65 mmHg
Heart rate	92 beats/min
Urinalysis	
Occult blood	(+1)
Renal ultrasound (left renal vein compression)	
Pre-stenotic diameter	0.91 cm
Stenotic diameter	∼0.14 cm
Diameter ratio	>3: 1
Contrast-enhanced cardiac CT (initial)	
Ascending aorta transverse diameter	∼4.5 cm (45 mm)
Aortic root anteroposterior diameter	3.6 cm

### Ancillary examinations

**Figure 2:**
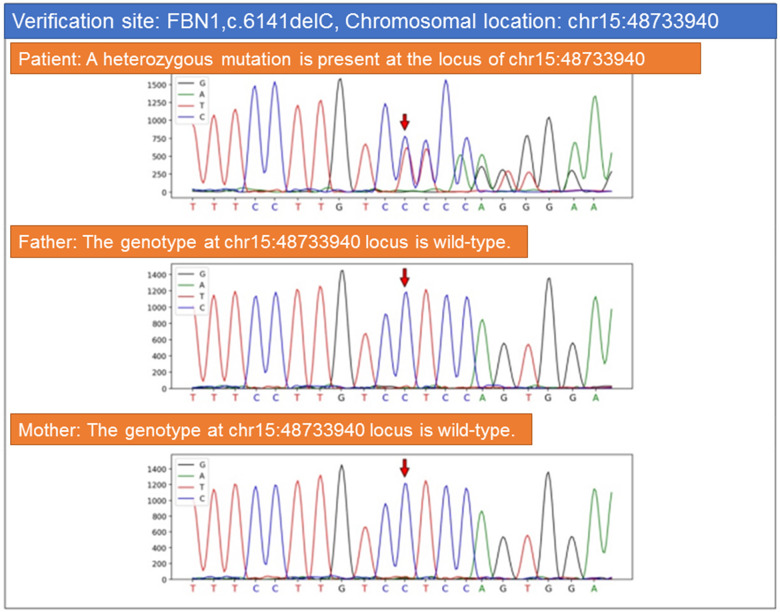
Genetic testing identified a heterozygous mutation in the FBN1 gene.

Urinalysis showed occult blood (+1). Serum troponin I was 0.001 ng/mL. Stool routine, high-sensitivity C-reactive protein, liver and renal function, electrolytes, myocardial enzymes, and coagulation profile were all within normal limits. Abdominal ultrasound was consistent with left renal vein compression. Electrocardiogram revealed sinus rhythm with atrial premature beats and incomplete right bundle branch block (Table 2). Echocardiography demonstrated: 1) mild left ventricular enlargement, dilated aortic sinus, and widened main pulmonary artery (aortic valve annulus ∼27 mm, sinus ∼43 mm, sinotubular junction ∼26 mm, proximal ascending aorta ∼27 mm; the aortic arch and descending portion were not dilated); 2) normal left ventricular systolic function. Contrast-enhanced cardiac CT demonstrated cardiomegaly, notably enlarged left and right ventricles, and dilatation of the ascending aorta with a transverse diameter of about 4.5 cm and an anteroposterior diameter of 3.6 cm at the root. Genetic testing identified a heterozygous mutation in the FBN1 gene, supporting the diagnosis of Marfan syndrome (Figure 2).

**Table 2:** Serial echocardiographic measurements of aortic root dimensions.

Date	Aortic valve annulus (mm)	Sinus (mm)	Sinotubular junction (mm)	Proximal ascending aorta (mm)
June 19, 2020 (initial admission)	∼27	∼43	∼26	∼27
February 10, 2022	∼27	∼45	∼30	∼27
January 12, 2023	∼26	∼47	∼29	∼29
July 19, 2023	∼26	∼46	∼29	∼28

### Diagnosis

(1). Marfan Syndrome. (2). Left Renal Vein Compression Syndrome.

### Management and follow-up

During hospitalization, the patient exhibited no significant cardiovascular symptoms. Regular outpatient follow-up was advised after discharge. Upon discharge, the patient was advised to undergo regular outpatient follow-up every 3 months, with routine echocardiographic monitoring of aortic root dimensions and left ventricular function. In addition, lifestyle modifications were recommended, including avoidance of strenuous physical activities and competitive sports to minimize cardiovascular stress. The patient and his family were also educated regarding the importance of blood pressure control and adherence to scheduled cardiology reviews.

### Echocardiography on February 10, 2022

1. Mild left ventricular enlargement and aortic sinus dilation (aortic valve annulus ∼27 mm, sinus ∼45 mm, sinotubular junction ∼30 mm, proximal ascending aorta ∼27 mm; aortic arch and descending portion not dilated);
2. Normal left ventricular systolic function.

### Echocardiography on January 12, 2023

1. Mild left ventricular enlargement and aortic sinus dilation (aortic valve annulus ∼26 mm, sinus ∼47 mm, sinotubular junction ∼29 mm, proximal ascending aorta ∼29 mm; aortic arch and descending portion not dilated);
2. Normal left ventricular systolic function.

In February 2023, the patient was readmitted due to “chest pain for 4 months.” Dynamic electrocardiography revealed frequent atrial and ventricular premature beats. Cardiac MRI showed left ventricular fullness, aortic sinus dilation, and evidence of myocardial fibrosis at the left ventricular base, consistent with early cardiac changes associated with Marfan syndrome. The patient was treated with metoprolol and propafenone hydrochloride for rhythm control, along with sodium creatine phosphate for myocardial support, resulting in symptom relief. After discharge, medication was continued as prescribed, with regular follow-up monitoring. History of metoprolol administration: On February 2, 2023, the patient started taking metoprolol sustained-release tablets (47.5 mg × 7 tablets per pack), one tablet once daily. The medication was discontinued in April 2023. On August 1, 2023, metoprolol (25 mg) was reinitiated at a dose of 1.25 tablets twice daily, and was discontinued on August 7, 2024.

### Echocardiography on July 19, 2023

1. Left ventricular internal diameter at the upper limit of normal and aortic sinus dilation (aortic valve annulus ∼26 mm, sinus ∼46 mm, sinotubular junction ∼29 mm, proximal ascending aorta ∼28 mm; aortic arch and descending portion not dilated);
2. Normal left ventricular systolic function.

## Discussion

In this study, a 13.5-year-old boy was admitted for excessive growth (18 cm in two years), visual impairment, and toe contractures. Examination revealed a tall, slender build with classic Marfan stigmata with a heterozygous FBN1 mutation. Investigations revealed frequent arrhythmias and cardiac MRI demonstrated myocardial fibrosis. Treatment with metoprolol and propafenone was initiated for rhythm control, resulting in symptom improvement. A concurrent diagnosis of left renal vein compression syndrome was also established. Management involves continuous beta-blocker therapy and regular cardiac surveillance to monitor aortic dimensions and ventricular function, consistent with standard Marfan syndrome care. MFS is an autosomal dominant connective tissue disorder affecting multiple systems, associated with mutations in genes such as FBN1 (encoding fibrillin-1) and those encoding transforming growth factor-beta (TGF-β) receptors (TGFBR1 or TGFBR2). Notably, a significant proportion of patient harbor mutations in the FBN1 gene (4). Classic manifestations of MFS include aortic root dilation, ectopia lentis, and skeletal abnormalities (5). Cardiovascular involvement is nearly universal in pediatric MFS patients, commonly presenting as mitral valve prolapse and aortic root dilation (6). In this case, the aortic root diameter reached 43 mm at the age of 13.5 years, which, to our knowledge, is relatively uncommon. According to the Z-score calculation formula by Pettersen et al. (7), an aortic root diameter of 43 mm significantly exceeds the predicted 99th percentile value for healthy adolescents. Such a large aortic root diameter is rare even among high-risk pediatric patients. Baseline data from Lacro et al. (8) on over 600 young MFS patients (mean age ∼11 years) reported an average maximum aortic root diameter of approximately 34.6 mm. The risk of aortic dissection and rupture progressively increases with aortic root dilation. Once the aortic root dilates to a certain threshold, prophylactic aortic root replacement surgery is indicated. However, current pediatric surgical criteria are largely extrapolated from adult guidelines. The European Reference Network for Rare Vascular Diseases (VASCERN) (9) recommends considering surgery in children when the aortic root diameter reaches 50 mm. Intervention at 45 mm may be considered in the presence of additional risk factors, such as a family history of aortic dissection at small diameters, a rapid annual growth rate (>5 mm/year and an increase in Z-score > + 1 SD), or concomitant need for valvular surgery. In our patient, the initial echocardiogram showed an aortic root diameter of 43 mm, and no specific intervention was undertaken at that time. The aortic root demonstrated an annual growth rate of approximately 3 mm/year. Metoprolol was later initiated due to concurrent premature beats. Currently, the patient remains asymptomatic.

In this case, it is important to distinguish between the nutcracker phenomenon and nutcracker syndrome. Nutcracker phenomenon refers to the anatomic compression of the left renal vein between the abdominal aorta and the superior mesenteric artery, which may be an incidental finding. Nutcracker syndrome is defined when this compression is accompanied by clinical symptoms such as hematuria, proteinuria, or left flank pain. Our patient presented with persistent occult hematuria and imaging evidence of left renal vein compression, fulfilling the criteria for nutcracker syndrome. In contrast, Marfan syndrome is a systemic connective tissue disorder caused by pathogenic variants in the FBN1 gene, with cardiovascular, ocular, and skeletal manifestations. The diagnosis of Marfan syndrome in this case was established using the revised Ghent criteria, which integrate systemic features, family history, and genetic testing. Notably, the patient had a systemic score ≥7 (including pectus carinatum, arachnodactyly, scoliosis, and reduced upper-to-lower segment ratio), aortic root dilation (Z-score ≥2), and a pathogenic FBN1 mutation. Although both conditions may present in tall, slender individuals, their underlying pathophysiology and management differ substantially. Recognizing the coexistence of these two entities is essential to avoid misattributing hematuria to other causes in patients with Marfan syndrome.

### Patient perspective

Nutcracker syndrome (NCS) refers to symptomatic compression of the left renal vein between the aorta and the superior mesenteric artery. Its clinical presentation is variable, often including hematuria, proteinuria, and left flank or pelvic pain (10). In our patient, persistent occult hematuria on urinalysis prompted further investigation. Renal ultrasound revealed narrowing of the left renal vein (diameter ∼0.14 cm) as it passed between the aorta and the superior mesenteric artery, with a pre-stenotic diameter of 0.91 cm, resulting in a diameter ratio greater than 3:1, consistent with left renal vein compression and confirming the diagnosis of NCS. Although MFS is a multisystem disorder, current diagnostic criteria for MFS (11) do not include NCS. Both MFS and NCS patients often share a tall, slender body habitus. However, a direct association remains unclear, as the available evidence is largely limited to case reports. Ichihara et al. (12) reported a case of NCS secondary to chronic aortic dissection, suggesting that NCS should be considered in the differential diagnosis of unexplained hematuria in patients with chronic aortic dissection.

Based on the findings derived from our study. Management recommendations for nutcracker syndrome and the potential role of the patient's lean habitus should be addressed. It should be acknowledged that management of nutcracker syndrome depends on symptom severity. In mild cases with intermittent hematuria and no significant complications, conservative management is often the first-line approach, particularly in children and adolescents. This includes weight gain in underweight individuals, as increased retroperitoneal fat may relieve compression of the left renal vein by providing cushioning between the aorta and the superior mesenteric artery. Our patient had a markedly lean habitus (height 188 cm, weight 53.3 kg, body mass index 15.1 kg/m^2^), which likely contributed to the nutcracker phenomenon. In such cases, nutritional support to achieve a healthy weight may alleviate the entrapment. Conservative measures also include avoidance of strenuous physical activity and regular monitoring of urinalysis and renal function. For patients with persistent or severe symptoms (e.g., intractable hematuria, flank pain, or renal impairment), more invasive interventions such as left renal vein transposition or endovascular stenting may be considered.

## Conclusion

We describe a case of MFS complicated by NCS, presenting with severe aortic dilation in childhood. Typical signs and cardiovascular abnormalities in pediatric MFS patients often emerge during adolescence. Early recognition and vigilant imaging surveillance are crucial for diagnosis and management.

## Data Availability

The raw data supporting the conclusions of this article will be made available by the authors, without undue reservation.
